# Antitumoral potency of methanolic extract from *Nitraria retusa* leaves via its immunomodulatory effect

**DOI:** 10.1186/s12935-015-0232-y

**Published:** 2015-08-25

**Authors:** Jihed Boubaker, Fadwa Chaabane, Ahmed Bedoui, Rihab Aloui, Besma Ben Ahmed, Kamel Ghedira, Leila Chekir-Ghedira

**Affiliations:** Laboratory of Cellular and Molecular Biology, Faculty of Dental Medicine, University of Monastir, Rue Avicenne, Monastir, 5000 Tunisia; Unity of Bioactive Natural Substances and Biotechnology, Faculty of Pharmacy, University of Monastir, Rue Avicenne, Monastir, 5000 Tunisia; Higher Institute of Medical Technologies of Tunis, Tunis El Manar University, Tunis Rommana, 1068 Tunisia

**Keywords:** *Nitraria retusa*, Melanoma, Antitumor, Immunomodulatory, Natural killer, Splenocyte

## Abstract

**Background:**

The purpose of this study was to assess the antitumoral effect of the methanol extract (MeOH) from *Nitraria retusa* leaves and to investigate its immunomodulatory activity that mediated the prevention of tumor progression in tumor-bearing mice.

**Methods:**

Balb/c mice weighing 18–20 g were subcutaneously implanted with B16-F10 cells then injected intra-peritoneally, 7 days later with (200 mg/kg bw) of MeOH extract, for 21 days. After euthanization on day 21, the tumors were weighed. Lymphocyte proliferation, cytotoxic T lymphocyte (CTL) and NK activity were evaluated using the MTT assay. Macrophage phagocytosis was studied by measuring their lysosomal activity and nitric oxide production.

**Results:**

The methanol extract inhibited significantly the growth of the implanted tumor, and increased remarkably splenocyte proliferation as well as NK and CTL activities, in tumor-bearing mice. It also promoted lysosomal activity of treated animal macrophages.

**Conclusion:**

Our findings suggest that antitumoral effect of MeOH extract is related with to immunomodulatory activity.

## Résumé

**Introduction:**

Le but de cette étude est d’évaluer l’effet antitumoral de l’extrait méthanolique (MeOH) issue des feuilles de *N. retusa* via son potentiel immunomodulateur et sa capacité à prévenir la progression tumoral chez des souris porteuses de tumeurs.

**Méthodes:**

Pour cette étude, nous avons implanté par voie sous-cutanée, chez des souris Balb/c de 18 à 20 g, des cellules B16-F10. Après apparition de la tumeur, au bout de 7jours de l’injection de B16-F10, nous avons entamé un traitement par injection intrapéritonéale de 200 mg/kg de poids corporel, d’extrait MeOH, et ce durant 21 jours. Les animaux sont euthanasiés au 21^ème^ jour, et les tumeurs sont pesées. La prolifération des splénocytes et des lymphocytes T cytotoxiques (CTL) à été évalué à l’aide du test au MTT. L’évaluation de l’activité NK à également été effectuée à l’aide du par le même test. Dans un second volet, nous avons étudié l’activité phagocytaire des macrophages en évaluant leur activité lysosomale et la production d’oxyde nitrique.

**Résultats:**

L’extrait MeOH révèle, un fort potentiel inhibiteur de la croissance de la tumeur transplantée, une augmentation de façon remarquable de la prolifération des splénocytes ainsi qu’une forte induction des activités NK et CTL chez des souris porteuses de tumeurs. Aussi l’extrait a considérablement induit l’activité phagocytaire des macrophages en augmentant leur activité lysosomale ainsi que la production de monoxide d’azote, par ces cellules.

**Conclusion:**

Nos résultats suggèrent fortement que l’effet antitumoral de l’extrait méthanolique passe par son potentiel immunomodulatrice.

## Background

Melanoma is the most aggressive form of skin cancer with an annual incidence consistently increasing worldwide [1]. Treatment options are limited for advanced stage patients and new therapies showing success in only a subset of patients [2].

The immune system is capable of recognizing melanoma tumors, and patients readily develop melanoma-specific T cell responses [3]. However, in most cases, these immune responses ultimately fail to eradicate established melanoma tumors [4].

Immunomodulation through natural or synthetic substances may be considered an alternative for the prevention and cure of neoplastic diseases [5]. The enhancement of host immune response has been recognized as a possible means of inhibiting tumor growth without harming the host [6]. Therefore, it is very important to investigate novel antitumor substances with improving immunity potential.

*Nitraria retusa* is commonly used in Tunisian traditional medicine to treat stomach pain, ulcers and gastritis. However, there are no studies regarding the antitumoral or immunomodulatory activities of *N. retusa*. Recently, we have demonstrated that methanol extract from *N. retusa* leaves exhibited antiproliferative potential against cancer cell lines in vitro [7]. Moreover, apoptotic effect of this plant has been carried out in some cancerous cell lines [7, 8]. However, there are no studies regarding the antitumoral or immunomodulatory activities of this plant.

Thus, we evaluated in this study the melanoma growth inhibiting capacity, of the MeOH extract against B16-F10 mouse melanoma cells, implanted in Balb/c mice in vivo. In addition, we investigated the potency of the tested extract on host immune responses of tumor-bearing mice.

This study show, for the first time, that methanolic extract of *N. retusa* leaves exert in vivo antitumoral and immunomodulatory activities.

## Results

### Phytochemical study of methanol extract from *N. retusa* leaves

As described in our previous study [7], methanol (MeOH) extract exhibited the highest quantities of flavonoids and polyphenols respectively (146.52 μg/ml concentration equivalent quercetin and 30 μg/ml concentration equivalent gallic acid).

### Cytotoxic effect on B16-F10 cell line

**Fig. 1:**
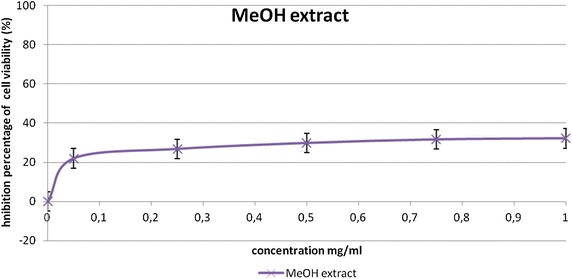
Anti-proliferative effect of methanol extract of *Nitraria retusa* leaf on B16-F10 melanoma cells. Values represent the mean ± SD of three separate experiments

We have examined the effect of different concentrations (ranging from 10 to 1000 μg/ml) of (MeOH) extract on B16-F10 cell growth using the MTT assay. The results of this assay are reported in (Fig. 1). MeOH extract inhibited the malignant tested cell growth.

### Acute toxicity

No mortality was recorded in the group of animals treated with the MeOH extract at 200 mg/kg BW.

### Effect of methanol extract on weight and volume of tumor

**Fig. 2:**
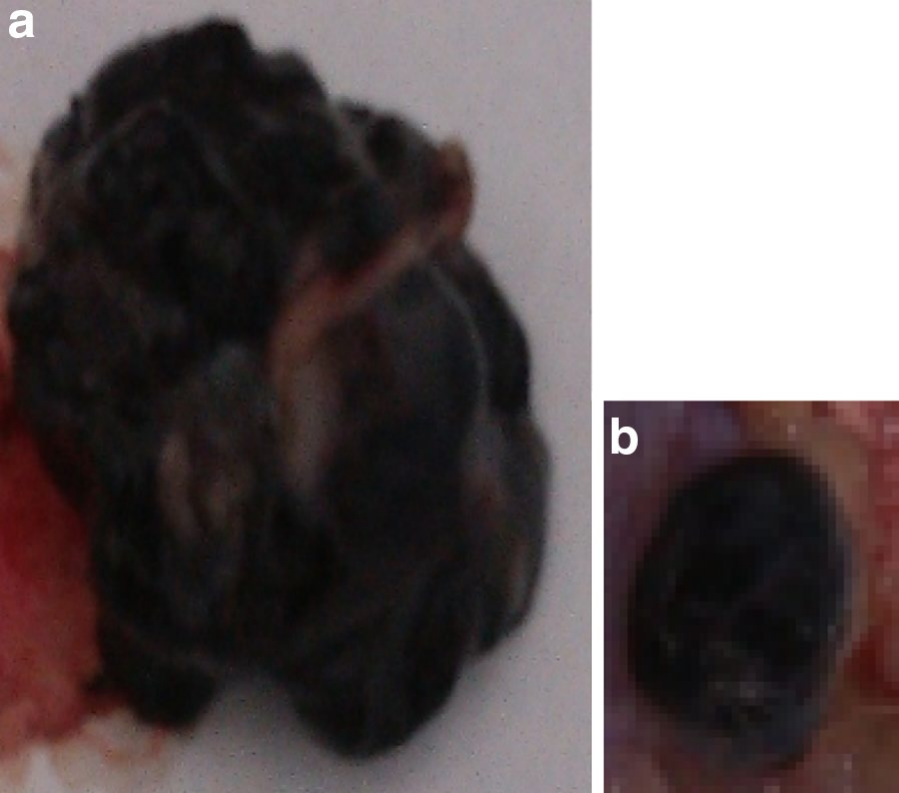
Inhibitory effect of *N. retusa* methanol extract on subcutaneous melanoma in Balb/c mice. Mice were sacrificed and then analyzed for representative macroscopic photographs of melanoma tumor weight and volume were determined. **a** Animal bearing tumor (positive control) (PC), **b** from animals bearing tumor and treated with MeOH extract at 200 mg/kg b.w

**Fig. 3:**
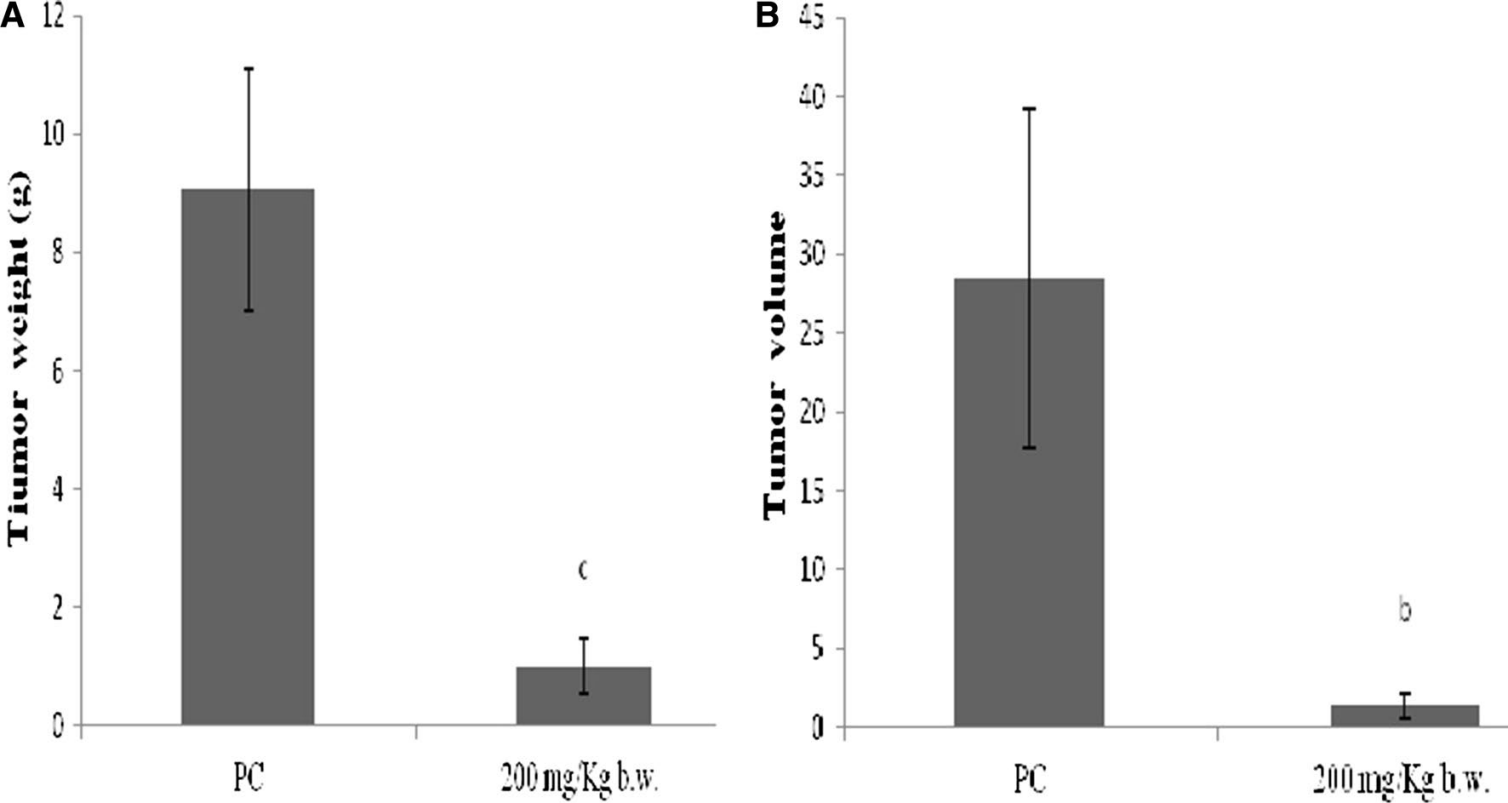
Effect of methanol extract from *N. retusa* on B16F10 melanoma growth in Balb/C mice. **A** Effect of tested extract on tumor weight, **B** effect of tested extract on tumor volume. All data shown are mean (±SD) percent inhibition values, and are representative of two independent experiments. (*b*) *P* < 0.01 significantly different from that of mice with melanoma (PC). (*c*) *P* < 0.001 significantly different from that of mice with melanoma (PC)

Our results suggest that (MeOH) extract significantly suppresses growth of tumor cells in the tumor-bearing mice. The average tumor size in (MeOH) extract-treated group was significantly smaller than that in the positive control group (Fig. 2). The inhibition of tumor volume reached 95.19 %. Consistent with this result, the average tumor weight after the administration to animals of the extract, was significantly lower than in positive control (animal with tumor) (PC). The inhibition percentage reached 88.95 % (Fig. 3).

### Effect of methanol extract on splenocytes proliferation

**Fig. 4:**
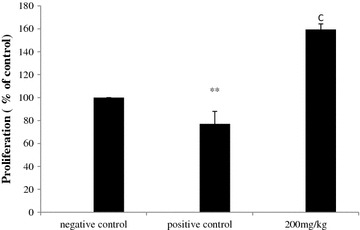
Effect of methanol extract from *N. retusa* on splenocytes proliferation in vivo. All values shown are mean (±SD) % activities relative to those of cells from PBS-only mice (non-tumor-injected; TN) at each time point, and representative of two independent experiments (*n* = 10 mice per group/time point). **P < 0.01 Significantly different from that of the control mice (NC). (*c*) p < 0.001 significantly different from that of mice with melanoma (PC)

The results showed that spontaneous proliferative capacity of splenic lymphocytes decreased in positive control mice (PC) compared to healthy mice (NC). Proliferative capacities decreased, after tumor induction, to 22.94 %. Treatment with methanol extract (200 mg/kg b.w) significantly restored the proliferation of splenic lymphocytes. The percentage of stimulation reached 64.2 % (Fig. 4).

### Effect of methanol extract on NK cell activity

**Fig. 5:**
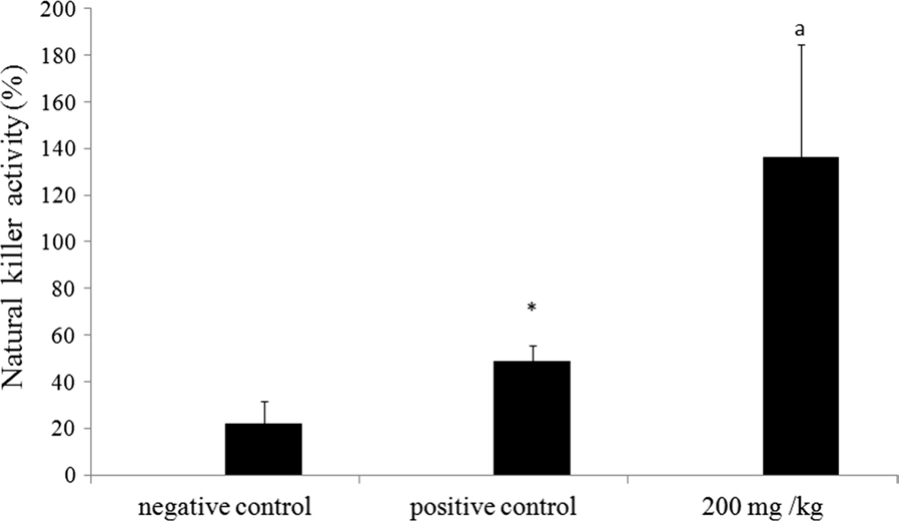
Effect of methanol extract from *N. retusa* on NK cell activity in vivo. All data shown are mean (±SD)  % NK activity, and are representative of two independent experiments (*n* = 10 mice per group/time point). *P < 0.05 significantly different from that of the control mice (NC). (*a*) *P* < 0.05 significantly different from that of mice with melanoma (PC)

The results of this assay were reported in Fig. 5. In fact, NK activity increases from 21.89 % in healthy mice to 48.66 % in the tumor-bearing (PC) mice. Treatment with (MeOH) extract increased the NK activity by 136.27 % in tumor-injected mice, revealing thus an induction of this cell defense system.

### Effect of methanol extract on CTL activity

**Fig. 6:**
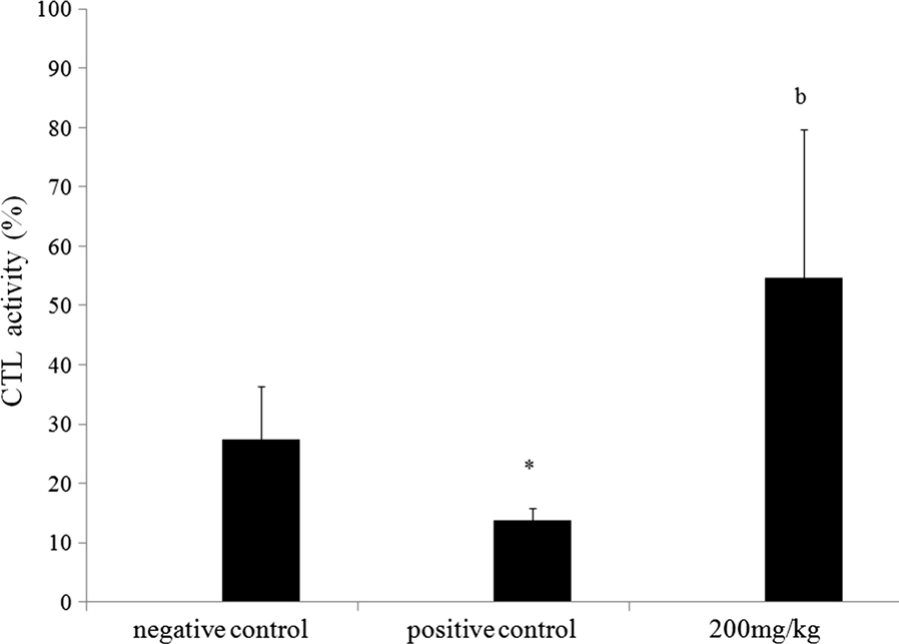
Effect of methanol extract from *N. retusa* on cytotoxic T-lymphocyte (CTL) activity in vivo. All data shown are mean (±SD) % killing activity, and are representative of two independent experiments (*n* = 10 mice per group/time point). *P < 0.05 significantly different from that of the control mice (NC). (*b*) *P* < 0.01 significantly different from that of mice with melanoma (PC)

Spleen cells from the experimental mice were assessed for specific cytotoxic activity against B16F10 melanoma cells (Fig. 6). Data reported in Fig. 5 showed that the presence of the tumor, significantly suppressed cytotoxic capacities of CTL. The CTL activity decreased from 27.33 % in the group of healthy mice (NC) to 13.77 % in the tumor-bearing mice group (PC). Treatment with MeOH extract allows rising of CTL activity, in the tumor-injected mice, to 54.64 % on days 21.

### Effect of methanol extract on peritoneal macrophage lysosomal activity

**Fig. 7:**
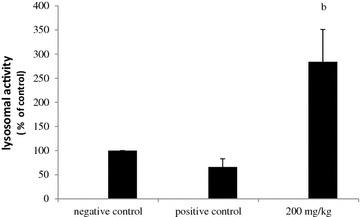
Effect of methanol extract from *N. retusa* on lysosomal activity of mouse peritoneal macrophages in vivo. All values shown are mean (±SD) % activities relative to those of cells from PBS-only mice (non-tumor-injected; TN) at each time point, and representative of two independent experiments (*n* = 10 mice per group/time point). (*b*) *P* < 0.01 significantly different from that of mice with melanoma (PC)

The activity in macrophages harvested from tumor-injected mice (PC) decreased significantly (by 65.87 %) when compared to untreated animals.Whereas lysosomal activity of macrophages obtained from MeOH extract treated tumor bearing mice (200 mg/kg b.w) increased by 255.37 % when compared to that of untreated mice cells (Fig. 7).

### Effect of methanol extract on peritoneal macrophage nitric oxide production

**Fig. 8:**
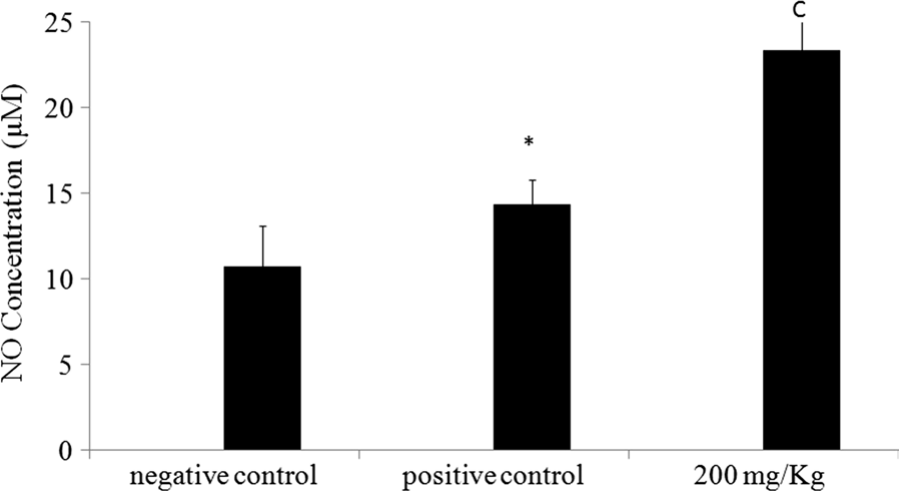
Effect of the methanol extract from *N. retusa* on production of the nitrite by mouse peritoneal macrophages in vivo. Data shown are mean (±SD) percentage of NO production from three independent experiments. *P < 0.05 significantly different from that of the control mice (NC). (*c*) P < 0.001 significantly different from that of mice with melanoma (PC)

Macrophages isolated from mice treated with MeOH extract (200 mg/kg b.w) for 24 h, display a significant increase in NO production. In fact, NO concentration rises from 14.38 µM (PC: positive control) to 23.35 µM (macrophage treated mice) Fig. 8

## Discussion

The number of new cancer cases each year is projected to rise worldwide by about 70 % by 2030 due to demographic changes alone, with the largest increases in the lower-income countries. Lung, liver, colorectal and breast cancers are the most commonly diagnosed in males and females respectively, and these cancers also represent the most frequent types of cancer related deaths. Thus, medical advances in disease treatment are an issue at a later stage and should be made available globally [9].

Our study revealed that MeOH extract inhibits melanoma cell proliferation in a dose dependent manner. This cytotoxicity may be ascribed to the presence of specific components such as polyphenols and flavonoids [7], as far as. They were the main constituents of the tested extract. In addition, Martinez et al. and Yanes et al. [10, 11] reported previously then antiproliferative activity of such compounds against melanoma cells. Our aim was to investigate alternative phytotherapy solutions to current anticancer treatments and preventing cancer development. Thus we demonstrated that *N. retusa* leaf extract exhibits an anti-tumoral potency in melanoma-bearing mice. Despite the fact that the mechanisms underlying MeOH extract anti-tumor potency may be complex, our data showed that this potency was most likely related to the regulation of the immune responses. Host immunity is involved in the development and progression of malignant disease. Some studies had reported that anti-tumor potencies of plant extracts should involve the enhancement of the main executors of the immune defense cells, such as lymphocytes and macrophages [12]. Thus, T cells can assign tumor cells directly or can act indirectly by releasing cytokines that amplify cytotoxic T lymphocyte responses or activate NK cells and macrophages [13]. In fact, CTL and NK cells are rich in cytoplasmic granules. Following degranulation, the cells produce specific biologically active substances, which have a cytotoxic activity on target cells. These granules contain perforin, granzymes, granulysin and other effector molecules involved in the anti-tumor potency, as well as some unidentified components [14, 15].

Our study revealed that treatment of tumor-bearing mice with MeOH extract increases their NK cells activity of. In fact, NK cell are known to be the major mediators of the innate anti-tumor immune responses; they eradicate tumors by recognizing stress-inducible ligands on tumors cells and execute tumor cells with perforin and granzyme in vivo [16]. NK cells stamp out solid tumors by apoptosis [16].

The proliferation of splenocytes induced by MeOH extract in tumor bearing mice could be attributed to flavonoids and polyphenols [17], a class of agents already known to be mechanistically active on immune system [18] and to modulate the immune activity [19].

One of the most important non-specific immune activities in situ, is phagocytosis performed by macrophages (as well as by other types of leukocytes). Phagocytosis is accompanied by the release of lysosomal enzymes like acid phosphatase (AP) used for the killing and digesting of microbial pathogens. The higher AP activity, the greater the phagocytic stimulation and intracellular killing capacity [20]. Our study showed that the tested extract increases significantly cellular lysosomal enzyme activity. This is most likely attributed to the flavonoid, tannin and polyphenol contents of the tested extract, which could be responsible for this particular immunomodulatory potency [18].

Macrophage phagocytosis is also assigned by the liberation of free radicals such as nitric oxide (NO), involved in pathogen killing [21]. We showed that MeOH extract induces NO synthesis, by peritoneal macrophages, reflecting phagocytic stimulation; as such, the extract could act as an immunostimulant of innate immunity. Many plants have been shown to affect lysosomal activity and NO synthase activity in macrophages [22, 23]. This result could be ascribed to the presence of polyphenols and flavonoids in methanol extract. Polyphenols are known to enhance the nitroblue tetrazolium (NBT) reducing activity of macrophages [24], to activate morphological change (spreading) of peritoneal macrophages, and to induce various antitumor cytokines including tumor necrosis factor (TNF), interleukin (IL)-1 [25], and nitric oxide [26].

It is noteworthy that development of melanoma is associated with immune suppression [27]. This is in accordance with our findings, which demonstrate a decrease in CTL activity in mice bearing-tumor but not those treated with the tested extract.

On the other hand, we observed a significant pro-apoptotic activity of the methanol extract against several as tumoral cell lines in vitro K562 [7], TK6 and B16f10 (data not shown), we assessed the ability of the extract to inhibit cell proliferation in vivo. This study showed that 200 mg/kg bw of MeOH extract reduced tumor size and weight. Such results were in agreement with findings of other researchers who reported the in vivo anti-tumoral activity of some plant extracts [12].

## Conclusion

Our results indicate for the first time, a significant anti-tumor effect of *N. retusa* leaf extract. This property is associated to immunomodulating effect upon splenocytes, NK cells and macrophages. However further characterization of bioactive compounds in the crude extract is warranted in future studies. The pharmacologically active ingredients and the signaling pathways involved in the antitumor activity also remain to be further elucidated.

## Methods

### Plant material

Leaves of *N. retusa* were collected from saline soils in Sahline, a region situated in mid-Tunisia, in December 2006. Their identification was done by Pr. M. Cheieb (Department of Botany, Faculty of Sciences, University of Sfax, Tunisia), according to the Flora of Tunisia [28, 29]. A voucher specimen (N.r-12.06) has been kept in our laboratory for future reference. The leaves were hade dried, powdered, and stored in a tightly closed container for further use.

### Preparation of methanolic extract

Three hundred and fifty grams of powder, from dried leaves, were sequentially extracted in a Soxhlet apparatus (6 h) (AM Glassware, Aberdeen, Scotland, United Kingdom) with hexane, chloroform, ethyl acetate and methanol solvents. We obtained the correspondent extracts for each solvent. Hexane (Hex), chloroform (Chl) and methanol (MeOH) extracts, with different polarities, were concentrated to dryness and the residues were kept at 4 °C. Then, each extract was resuspended in dimethyl sulfoxide solvent (DMSO). Plant materials were screened for the presence of tannins, flavonoids, coumarins and sterols using the methods previously described by Boubaker, et al. [30]. Amongst these extracts, only the methanolic one was used in the evaluation of the in vivo antitumoral and immumodulatory effects.

### Cell line and culture

The B16F10 melanoma line was obtained from American Type Culture Collection (ATCC, Manassas, VA, USA) and grown at 37 °C in a humidified incubator with 5 % CO_2_ at 37 °C. The cells were cultured in RPMI-1640 medium supplemented with 10 % (v/v) fetal calf serum (FCS, Biowhitaker, Lonza, Belgium), 2 mM glutamine, 1 % NEA (100X), 1 % sodium pyruvate 100 mM (complete RPMI).

### Assay for cytotoxic activity

Cytotoxicity of *N. retusa* extracts against B16F10 cells was estimated by the 3-(4,5-dimethylthiazol-2-yl)-2,5-diphenyltetrazolium bromide (MTT) assay, based on the reduction of the MTT by mitochondrial dehydrogenases in viable cells. The resulting blue formazan product is measured spectrophotometrically [31] Cells were seeded in a 96-well plate at a concentration of 5 × 10^3^ cells/well and incubated at 37 °C for 24 h in a 5 % CO_2_ enriched atmosphere. The extract was firstly dissolved in 1 % DMSO, then in the cell growth medium. Cells were incubated again at 37 °C for 48 h with each of the tested extract at concentrations ranging from 10 to 1000 μg/ml. Next, the medium was removed and cells in each well were incubated with 50 μl of MTT solution (5 mg/ml) at 37 °C for 4 h. MTT solution was then discarded and 50 μl of 100 % DMSO were added to dissolve the insoluble formazan crystal. The optical density was measured at 540 nm. Each drug concentration was tested in triplicate.

The cytotoxic effects of the extract was estimated in terms of cell population growth inhibition percentage and expressed as IC_50_ which is the concentration of extract that reduces the absorbance of the treated cells by 50 % with reference to the control (cells treated with DMSO). The IC_50_ value was graphically obtained from the dose–response curves.

### Experimental animals

Specific pathogen-free Balb/c mice (6–8 week-old males, 20–25 g) were obtained from the Pasteur Institute (Tunis, Tunisia). The animals were housed in polypropylene cages with stainless steel grill tops and provided with bedding of clean paddy husk. The animals were acclimatized to laboratory conditions for one week prior to treatment. All mice were housed under standard conditions of temperature (25 °C), humidity (60 %), and light (12-h light/dark) in an accredited pathogen-free facility. The mice were fed with standard laboratory pelleted feed. All animal experiments were performed in accordance with the guidelines for the care and use of laboratory animals published by the US National Institutes of Health. The study protocol was approved by the Ethics Committee of the University Hospital Fattouma-Bourguiba of Monastir, Tunisia.

### Subcutaneous melanoma model in vivo treatment

Mice were separated into three experimental groups (n = 60; two separate studies using n = 10/group): mice in the first group were treated with intraperitoneal injection of MeOH at 200 mg/kg BW starting immediately after seven days of a single subcutaneous (SC) injection of 2 × 10^6^ viable B16F10 tumor cells into their right hind leg (thigh) on Day 0. The second group was comprised of B16F10-injected mice that were treated only with PBS (PC: positive control) throughout the post-injection period. The last group (NC: negative control)—‘healthy mice’—received no tumor cells but received injections of PBS only in the post-injection period. The processing time of treatment period was for 21 days and every 2 days.

On Day 21, all mice were weighed and sacrificed. Solid tumors were collected and weighed, the longest and shortest diameters of tumors were measured with a vernier caliper and the tumor volume was determined according to established procedure [32]: Tumor volume (mm^3^) = (L × W^2^) × 0.5 and then the inhibitory rate against tumor growth was calculated as (%) = [(PC − Trt)/PC] × 100 %, where PC is the average tumor weight in the tumor control mice (“PC” group above) and Trt is the weight in mice that had received methanolic extract.

### Acute toxicity

For acute toxicity, mice were divided into two groups of six animals each. The first group served as a control and received only the vehicle solution, while the second group was treated by intraperitoneal injection (i.p) with dose of 200 mg/kg b.w. of MeOH extract.

### Splenocytes proliferation assay

Mice were killed on day 21, and the spleen of each mouse was isolated aseptically and minced using a sterile forceps. The splenocytes were then centrifuged (1500 rpm, 10 min) and any red blood cells present lysed by re-suspending the pellet in lysing buffer (144 mM NH_4_Cl, 1.7 mM Tris-Base) and placing on ice for 10 min. Cells were then washed twice with PBS and then re-suspended in complete RPMI. Splenocytes proliferation assays were performed by using the MTT method. Splenocytes suspensions in complete RPMI (5 × 10^5^ cells/well) were incubated in 96-well plates for 48 h at 37 °C. Thereafter, the plates were centrifuged at 1500 rpm for 10 min, then each cell pellet was resuspended in 50 µl MTT (5 mg/ml) in RPMI. The plate was then incubated a further 2 h at 37 °C before the plates were centrifuged again, the MTT solution in each well was removed, and 100 µl DMSO were added to each well. Percentage proliferation was calculated as:

$${\text{Proliferation }}\left( \% \right) \, =\,{ 1}00 \, \times \, \left( {{\text{OD sample}} - {\text{OD NC}}} \right)/{\text{OD NC}})$$

[33].

### Cytotoxic activity of natural killer (NK) cells

Splenocytes isolated from each mouse were incubated with K562 cells. Briefly, splenocytes (5 × 10^6^ cells/well) (effector cell) were seeded in the well with the K562 cells (5 × 10^4^ cells/well) (targeted cell). The plates were incubated at 37 °C with 5 % CO_2_ for 4 h, and 50 µl MTT solution (5 mg/ml) were added to each well. The plate were incubated a further 2 h and subjected to the protocols outlined above to assess formazan formation. NK cell activity was calculated as: NK activity (%) = 100 × (ODT − (ODS − ODE))/ODT) where ODT, optical density value of target cells control, ODS, optical density value of test sample, ODE, optical density value of effector cells control [34]

### Assay of cytotoxic T-lymphocyte (CTL) activity

Briefly, B16-F10 cells and isolated splenocytes were used as target and effector cells, respectively. The assay was performed as outlined above for the NK assay except that cells were incubated for 24 h. 50 µl MTT solution (5 mg/ml) were added to each well and the plate was incubated a further 2 h and subjected to the protocols outlined above to assess formazan formation. The percentage of target cells killed was determined as in the NK assay:

$${\text{CTL activity }}\left( \% \right) \, =\, { 1}00 \, \times \, \left( {{\text{ODT}} - \, \left( {{\text{ODS }} - {\text{ ODE}}} \right)} \right)/{\text{ODT}},$$

where ODT, optical density value of target cells control, ODS, optical density value of test sample, ODE, optical density value of effector cells control.

### Cellular lysosomal enzyme activity

Peritoneal macrophages were obtained after intraperitoneal injection of 5 ml sterile PBS, and withdrawal of the fluid. Obtained cells were washed twice by PBS and re-suspended in complete RPMI 1640; cell viability was assessed via trypan blue exclusion. One hundred µl of the macrophage cell suspension (6 × 10^6^ cells/ml) were seeded in 96 flat-botton well plates treated with different concentrations of extract and incubated for 24 h at 37 °C, under a humid atmosphere and at 5 % CO_2_. The adherent cells are taken up in 20 µl Triton X-100 (1 %), 10 µl of p-nitrophenyl phosphate (p-NPP) (100 mM), the acid phosphatase substrate, and 50 µl of citrate buffer (0.1 M, pH = 5). The whole is incubated at 37 °C in a humid atmosphere and 5 % CO_2_ for 30 min before 150 µl of borate buffer (pH 9.8, 0.2 M) was added to each well, and the absorbance measured at 405 nm. The percentage of the activity is determined by the following formula: Activity (%) = 100 × (OD sample-OD control)/OD control [35].

### Nitric oxide production

The amount of NO released by macrophages was measured by determining the amounts of accumulated nitrite (NO_2_) in cell free supernatants via the Griess reaction [36]. In brief, isolated macrophages of each group were incubated for 24 h. Nitrite was then measured by adding 100 µl Griess reagent (1 % sulfanilamide and 0.1 % naphthylenediamine in 5 % phosphoric acid) to 100 µl of harvested culture supernatant. The optical density at 570 nm (OD_570_) was then measured in a microplate reader (Thermo Scientific, Vantaa, Finland). NO concentrations were calculated by comparison with the OD_570_ of a standard solution of sodium nitrite diluted in culture medium and placed in parallel wells in the assay plates.

### Statistical analysis

Data were collected and expressed as the mean ± standard deviation of three independent experiments and analyzed for statistical significance from control. The data were tested for statistical differences by one-way ANOVA followed by student test using statistica. The criterion for significance was set at *P* < 0.05.

## Abbreviations

MeOH: methanol
DMSO: dimethyl sulfoxide
NK: natural killer
CTL: cytotoxic T-lymphocyte
NO: nitric oxide
